# Obesrvations on Diphtheria

**Published:** 1859-08

**Authors:** Stephen Monckton


### SELECTIONS.

DIPHTHERIA.—I. *Observations on Diphtheria.* By STEPHEN MONCKTON, M. D.

1. Diphtheria is a distinct disease, easily recognised, and not to be dreaded till such changes have occurred about the fauces and tonsils, as it is impossible to overlook. A remote kinship there certainly is between it and scarlet fever, but identical they are not; about here, as before mentioned, they have coëxisted largely; and in the same little hamlet children have been lying dead at the same time, some of scarlatina and some of diphtheria, not very many yards apart; but still it has never been for a moment difficult to determine of any individual case to which category it belonged; three times, at least, I have seen diphtheria in individuals where scarlet fever had been previously suffered. Again, though as the diphtheritic membrane loosens and separates from the surface of the throat and tonsil, sloughing ulceration *may* ensue, I feel at present fully persuaded that diphtheria and cynanche maligna are not the same thing.

2. Premonitory symptoms are often absent, and so far as I have seen, always very mild, *i. e* the first visible deposition of patchy white putty-form membrane is not preceded by acute fever, sore throat, prostration, or marked constitutional symptoms. Immediately after a suspicion has arisen that the patient ails, a spot as of soft Pâte de Guimauve, and more or less extensive, becomes distinctly visible about the isthmus faucium, most frequently, I think, occupying the sulcus between the anterior pillar and the tonsil; neither patient nor practitioner, therefore, need timidly apprehend the existence of this malady from either moderate or severe symptoms or disturbances of any sort: in real diphtheria the first suspicions are in a very few hours (from seven or eight to twenty-four) confirmed by the appearance of undoubted deposit in the throat.

3. The order of the appearance in the local phenomena seems to be this—first, upon the mucous surface is formed a soft, reasonable coherent cake of epithelial cells, which quickly extends in dimensions, and rather solidifies in substance; underneath this there is not at first ulceration or breach of surface, the cake consists wholly of epithelial cells without pus or fibres, and if removed leaves behind it a raw but unbroken basement membrane rather shining, and with bloody points, exceedingly like the skin from which an eczematous scab has been prematurely torn; this is best seen in cases where a large thick patch has formed upon the under surface of the velum, and begins to separate by a thick crescentic margin across the back of the palate about the eighth or ninth day. It would seem, however, that before the separation by natural process takes place, ulceration does begin, and with it some change in the character of the false membrane; as the glands often, but not always, enlarge, a few pus globules can be found in the deposit, and some distinct sloughy shreds of basement membrane are to be distinguished; but even when glandular swelling constitutes a painful and prominent feature, the ulceration and sloughing, as I have proved after death, is on a very limited scale.

4. Besides the specially characteristic false membrane, other points are to be noticed; the cervical glands commonly begin to enlarge about the fourth day, and occasionally swell to a very considerable extent, rendering it difficult to medicate the throat topically, or to get down the proper amount of medicine and food. I have also seen two deaths in which the fatal event did seem to be so largely resulting from obstructed breathing, as to bring me to the very eve of tracheotomising; but in one of these cases a *post-mortem* enabled us to attribute much of the dyspnœa to a clearly-developed, but thin, layer of the false membrane down larynx, windpipe, and larger bronchi. As a general rule, I should say that the glands swell in diphtheria more constantly, and less considerably than in scarlet fever. The adjacent tract of mucous membrane is also disordered, tongue foul, but not the clear white coat and punctuation of scarlet fever; nose full and tumid, and sometimes a slight acrid discharge from the nostrils is to be observed.

5. The constitutional symptoms at first altogether slight (the pyrexia, headache and sickness of early scarlatina being altogether wanting), become very real as the disease advances. The main feature is prostration; not typhoid at all, no coma, no sensorial disturbance throughout, no sordes, no heavy lurid look, no suggestion of a frame burdened with an animal poison; and in many cases the practitioner, if not warned by previous experience, *or a careful observation of the pulse*, is surprised to learn that the patient he left with clear countenance, cheerful manner, and little suffering a few hours ago, has just gone off, while casually sitting upright, in a fatal syncope. The theory seems almost warranted, that, whereas in typhus a poisonous nescio quid is gendered in the blood, we have here by the growth of the false membrane, or in some other way, an essential ingredient exhausted from it, precisely as the atoms constituting the difference between sugar and spirit are removed by the growth and vegetation of the yeast plant. The practical fact is, however, this, that after the fourth or fifth day a diphtheritic patient becomes the subject of very real asthenia, not so much perceived by the patient, as discoverable by the lax pupil and feeble pulse, and that this state is the one which about the eighth day is too apt to terminate in death. I have sometimes found albumen in the urine—sometimes not; more frequentiy in the late stages than the earlier ones.

9. *Treatment.*—Directly patches appear in the throat destroy them with caustic, and, despite biting, scratching, shouting, or remonstrance let there be no mistake about the *fortiter in re.* I can hardly conceive that for this purpose anything can equal the solid nitrate of silver. In the majority of instances it would be right to administer with your own hand at the same time a large dose of calomel. There is no depression now, and the amount of that which is to come will depend on the progress of the throat changes, which the caustic and calomel are designed to arrest. One more vigorous application of the caustic will be doubtless needed in from twelve to twenty-four hours; after that, unless the disease spreads around, discontinue it for a time; and should some large and tender glands begin to show, pencil them over freely on the outside with nitrate of silver; it is a quick, certain, manageable, counter-irritant, less annoying and depressing than a blister. Follow the first dose of calomel with aperients till the evacuations be satisfactory as to frequency and character. The throat mischief often yields as the edges of the tongue clean, and attention of this sort is, therefore, not to be dispensed with. Supply the patient with fresh air, abundant food and stimulus, and, especially after the sixth day, *keep him flat.*

As regards medicine, I give either muriatic acid, with chloric æther, every few hours, or pretty full doses of ammon. sesquicarb.—*Medical Times and Gazette.*

II. *Clinical Lecture on Diphtheria.* By DR. CORRIGAN.

[*The Dublin Hospital Gazette* (Feb. 15, 1859), contains a lecture founded on a fatal case of *diphtheria*, the concluding portion of which is subjoined :] On the *post mortem* examination, the appearances were such as to leave no doubt as to the nature of the disease. The root of the tongue, the soft palate, arches of the palate, the uvula on both surfaces, and the back of the pharynx, were covered with the pasty exudation described. It did not extend upwards into the nasal fossæ nor downwards into the œsophagus. The tonsils and lymphatic glands were unaffected. There was no appearance of gangrene, and the mucous membrane underneath was merely red and congested.—The epiglottis was not swollen. There was no sub-mucous inflammation—at least none worth noticing—but the pasty exudation was thick and jagged on the mucous membrane. The exudation did not extend into the larynx. The chordæ-vocales were slightly vascular, but otherwise free, as likewise were the ventricles of the larynx.

The two important questions now before us are—Is this a new disease? and if so, how are we to distinguish it from other affections of the throat which resemble it? On the answers to these questions, or on accurate diagnosis of it, must rest, as the only sure foundation, our after observation as to treatment.

I believe it is a new disease in this sense, that whether scattered instances of it have or have not occurred previously, its visitation as an endemic is new to us; and all that we know of it is sufficient to stimulate us to lose no time acquiring as rapidly as possible an intimate knowledge of it.

The first point to which I wish to draw your attention is the peculiar oval swelling of the neck, not extending to fill up the angles of the jaw. When the post-mortem was made, the deep-seated muscular tissues were found infiltrated with a yellowish gelatinous serum, and this infiltration will explain the peculiar shape of the swelling, when we bear in mind the anatomy of the cervical fascia. The inner layer of this fascia, dipping from the anterior edge of the sterno-cleido-mastoid muscle, bends inwards as it ascends, until it reaches the styloid process and inner angle of the jaw, and will give to an infiltration under it the oval form of the tumefaction, as presented in this case; while in the effusion accompanying scarlatina, the fluid is poured out equally under the subcutaneous layer of the fascia, which spreads upwards, to be lost in the skin and cellular tissue of the cheek, and thus gives the collared or rounded swelling to the jaws so characteristic of scarlatina. That the disease was not scarlatina was also sufficiently evident from the number of days that elapsed before the throat became engaged.—We must therefore dismiss all idea of this disease being scarlatina throat of any kind. The perfect freedom of the tonsils from all disease removes it equally from tonsillitis and all its subdivisions. Croup has occasionally attacked adults; I am not aware that it has ever attacked persons of advanced life; but the freedom of the trachea and larynx from disease distinguishes it from that malady.

Acute laryngitis in the adult is a very rapid disease, but neither in symptoms nor in post-mortem appearance does it even nearly resemble that disease. There was no whispering voice, no struggles for breath, no congested countenance, no staring eyeball, such as we see in acute laryngitis presenting mechanical obstruction to the passage of air, nor, on examination after death, was the larynx affected to any degree worth noticing. In acute laryngitis too, when the epiglottis is affected, it is with sub-mucous effusion, not, as in this case, with pasty exudation on both its surfaces.

Reviewing, then, the whole case in its symptoms and pathology, we must, I think, look upon the case before us as diphtheria, and practically as a new and terrible disease.

There still remains an important question for us; was the fever under which F. labored for several days one peculiar to diphtheria, or was it merely our non-maculated typhus—diphtheria terminating life, as an accompaniment or sequelæ—as we frequently see occur in other instances, whatever local disease is prevalent, having a tendency to be developed towards the termination of any of our depressing fevers? The latter was probably the case in the instance before us, but this cannot diminish our fears about the disease—whether as an idiopathic disease or as a sequela of fever, there is much to be dreaded about it.

On treatment I do not at present intend to make almost any observation. The indications clearly were, up to this period of the local affection of the throat, to treat the attack as one of adynamic fever.—Other observations for treatment must be reserved for future cases, of which I fear we shall have more than we desire, for our worst epidemics are generally ushered in by dropping cases, such as this. As to the influence of bad food, bad air, or contagion in generating this disease, I have to observe that this man was well fed, being in the police force, that he lived in a high and healthy part of the city, and in the part of the city most remote from all communication with shipping, or with persons passing through from England.

This accords with the circumstances of the first case of cholera which appeared in Dublin in the last outbreak, in 1849-50. The first case occurred in the Convict Depot, in Smithfield, in an inmate that had been in the depot for months, and barred in from all the possibilities that could support the idea of contagion. That case is given in the report of the Central Board of Ireland on the epidemic fever and cholera of that period.

It is better for the advancement of medical knowledge that we should admit we know little or nothing of the propagating causes of epidemics, than impede further research by advancing ill-founded hypotheses, and call them scientific theories.

### III. *Report of the Lancet Sanitary Commission on Diphtheria: Its History, Progress, Symptoms, and Treatment.*

Three distinct forms have prevailed in this country of diphtheric angina, or more briefly, of diphtheria. The first may be properly called simple diphtheric angina, or simple diphtheria; the second, croupal diphtheric angina, or croupal diphtheria; the third, malignant diphtheric angina, or malignant diphtheria.

1. Simple diphtheria is the mildest and most frequent form of the disease. It is preceded by more or less of fever, and by headache; the tongue is coated by a thick creamy deposit; some discomfort is complained of in the fauces, perhaps a slight difficulty in deglutition. It is usually at this time that the medical man has the opportunity of seeing the throat, and now (from twelve to thirty-six hours after the first invasion) one tonsil—rarely both—is covered by a small patch of white membranous deposit. This may extend and cover the whole of the soft palate, and the pharynx but *rarely*. It commonly, in this form of the affection, remains stationary, or extends but little; it does not blacken or putrefy, neither does it exhale the fœtid odor of putrescence. The surrounding mucous membrane is swollen, purple, and projecting; the subjacent tissue not uncommonly betrays a breach of surface, partly due to the injurious surrounding pressure. The submaxillary glands are somewhat tumefied, but neither the parotid nor the cervical glands are implicated.

The duration of this affection varies from five to nine days. It has been observed in nearly every district where the diphtheric type has shown itself. The prognosis is favorable. The treatment which succeeds best is the local application of a solution of nitrate of silver, thirty grains to the ounce, and the ferrochloric mixture, containing the tincture of sesquichloride of iron, in combination with chlorate of potash, with a judicious and sparing use of evacuants.

2. Croupal diphtheria, or croupal diphtheric angina, is a more severe manifestation of the diphtheric type, and is undoubtedly that by which the greatest number of deaths have been occasioned in this country. It is more frequent in children than in adults. Its precurrent symptoms are active fever, intense headache, hot skin, engorgement of the glands behind the jaw, and perceptible difficulty of deglutition. The parents are only now aroused to the existence of a morbid condition. When the surgeon is summoned, he finds the throat and mouth covered with yellow or brownish leathery exudation. Within a few hours a hoarse, barking cough, and a change in the tone of the voice are marked; oppression of the breathing supervenes; then paroxysms of suffocation, more and more frequent; the cough is stifled, and the voice also dies out. As the access of suffocation is felt, the poor child turns from side to side, throws its arms into the air, clutches its mother violently, and struggles furiously to gain breath, then falls exhausted in the bed, and gaining strength from momentary repose, renews the hopeless struggle to the end. Pehaps in a violent fit of coughing, it expels a false membrane from the air-tubes, which has extended down to the fifth division of the bronchi; then it breathes easily, smiles again, and sleeps, but soon wakes to resume its struggle with death—it may be again to expel the membrane, and finally to triumph. But such a happy victory is wholly exceptional, and when once the grip of the disease has closed upon the air-tubes, death claims its prey.

The drama is of another, but not less tragic character, if the sufferer be an adult. It is more prolonged, for the larynx and tracheal tube are more capacious, and the membrane advances far down the bronchi before the scene closees and the black curtain drops that shuts out the future from the gaze of straining human eyes. As the oppression of breathing, the piping tone of the voice, the stifled cough, and the agony of suffocation accede, the patient fights against them with all the energy which the intelligent perception of danger, the earnest desire for life, and the despairing sense of approaching fate can inspire. He has an heroic endurance, and does not murmur at the most barbarous cauterizations, if they afford only a temporary relief; nor must the surgeon shrink from them, but with deliberate and benevolent cruelty, thrusting back the epiglottis with a spatula, he must follow the disease into its home in the larynx and freely brush the surface with effective solutions of nitrate of silver, or of hydro-chloric acid.

As to the treatment of this croupal variety, empiricism has led to results widely differing from those which rational medicine had counselled. It is a disease characterized by rapid and excessive exudation. Therefore rational medicine counselled every form of antiphlogistic medicine. Calomel, leeches, phlebotomy, and blisters, have been lavished on the sufferers with an unfortunate prodigality. When these failed, the alkaline remedies were strongly counnselled, and especially the bicarbonate of soda. M. Marchal de Calvi and M. Lamaire contested the honor of recommending it.* Others have advocated with equal warmth the employment of full doses of bicarbonate of potash.† But these remedies, having been fully tested, may be pronounced useless here, or of exceptional utility only. "La rationalisme," says Trousseau in an admirable series of papers on this subject, which we have consulted with great interest and profit, "ne conduit en medecine qu'a des sottises." Croupal diphtheria is, indeed a phlegmasia, and in employing antiphlogistic remedies, you would arrive at results parallel to those of that unfortunate physician of Chapelle Verous, who lost sixty patients out of sixty cases which he treated wlth blisters, leeches, and bleeding—bleeding, blisters and leeches. Thus, Mr. Stiles, of Pinchbeck, who, in the short space of sixteen days, had not less than three hundeed diphtheric patients under his care (and to whom we are indebted for a very important study of the epidemic, which he witnessed, and by his intelligent efforts succeeded in greatly ameliorating), had the annoyance, in two out of the only three cases in which he was induced to apply blisters, to see the vesicated surfaces quickly covered with a diphtheric deposit, followed by sloughing; and thus, also, in the epidemic at Launcerton, Mr. Thompson found that blistered surfaces were attacked with diphtheric inflamation. So also with leech-bites.

Clinical experience teaches that the first importance is to be attached, in these cases, to local applications. The choice involves the exercise of judicious discretion. Unable to discuss here at length the cases on which we found our practice, we are compelled to adopt a mild dogmatism. In the earliest stage, a solution of nitrate of silver should be employed, of the strength of thirty grains to the ounce of distilled water. This is best applied to adults by the curved whalebone probang and sponge now in general use, and to the child with a full-sized camel-hair brush. For the adult, Dr. Richard Quain's tongue-depressor should be employed, if at hand, or the handle of a tea-spoon. In the case of a child, he should be placed on the knee of an attendant —not the mother—and the head fixed. If he will not open the mouth —and gentleness is very desirable—the nostrils should be closed for a moment; as he opens the mouth for breath, the jaw should be firmly

*The priority appears to be with M. Lemaire. See his paper "On the Employment of Bicarbonate of Soda as an Antiphlogistic," in the "Lancette Francise," vol. xii., p. 1416.

† See fnrther Marchal de Calvi, "L'Union Medicale," 1855 Dr. Lasegue, "Theses de Paris," 1856.

depressed, the lower lip being folded over the teeth to prevent the operator from being bitten, and then the tongue being kept down, the whole of the fauces are fairly brought into view, and may be thoroughly washed with the solution. This is no unimportant detail, for upon the effectual accomplishment of this manœuvre, the success of the treatment will greatly depend. It should be repeated three or four times in the twenty-four hours.

If the exudation continues to extend, a collutatory of hydrochloric acid should be applied in like manner, in the case of an adult. With children the addition of honey to the hydrochloric acid is desirable.—But this is not only a much more painful application than the solution of nitrate of silver, but it has the disadvantage of creating a superficial eschar which simulates the diphtheric exudation, and hinders the perception of the progress of the disease. The solid nitrate of silver labors under similar defects, and has a danger of its own, from the risk of a fracture of the pencil in the mouth, which if swallowed, might be fatal—an accident which has happened, and which has come near to such a result in more than one case.

The constitutional treatment in croupal diphtheria should commence with the employment of an active emetic; it matters little of what nature, so that it be immediate in its action. Ipecacuanha is to be preferred, as being less depressing than most others. If the symptoms should give evidence of arrest, the ferrochloric mixture may be employed with confidence. The valuable influence of the tincture of sesquichloride of iron has been fully tested in the English epidemics. Not only do Dr. Heslop, of Birmington; Dr. Kingsford, of Boston; Mr. Stiles, of Pinchbeck, and other competent observers of extensive epidemics, speak in the most confident terms of its value, but we have confirmation on every side of the results which they announce. The combination of chlorate of potash and hydrochloric acid, with the tincture of sesquichloride of iron, is strongly to be recommended, especially in these croupal cases the chlorate of potash having an undoubtedly anti-diphtheric influence, where time exists to bring it into play.

When, in spite of these measures, the diphtheric inflammation, traveling onwards, reaches the larynx, when the altered voice, the oppressed breathing, and the stifled and less frequent cough give warning that the vocal chords themselves are affected by the exudation, in spite of courageous and energetic cauterization, then the question of tracheotomy must be entertained. For us this is a settled point. There is no longer any need to recapitulate the arguments on the one side or the other: tracheotomy is a resource which surgery is bound to employ for the salvation of the patient under such circumstances, and in the view of what experience teaches is otherwise certain death. The statistics of tracheotomy at the Hôpital des Enfans in 1855, showed ten cures and thirty-eight deaths out of forty-eight cases, or one patient saved in five. But the statistics vary with the character of the epidemic, and with the courage of the surgeon who is so wise to counsel or to perform the operation when the indications we have given are established, or hesitates until the most valuable time is lost, and eternity is at hand for his patient. We would give only one counsel as to the performance of the operation : it is, that the trachea should be fixed with an ordinary tenaculum, or one grooved on its convexity.—It facilitates the operation by rendering it more secure and speedy. The most important precautions are those connected with the subsequent care of the patient, which includes the employment of a double tube, of which the inner may be frequently removed and cleansed ; the placing a light gauze around the neck ; the impregnation of the air with the vapor of water (from a kettle or otherwise) ; and the cauterization of the edges of the wound.

III. Malignant diphtheria, or malignant diphtheric angina, is that form which has attracted the most careful attention, and has impressed medical observers in this country most strongly with the active and fatal character of the diphtheric poison. We have ample materials for the description of this most severe form. Its prodromata are, intense headache, severe febrile condition, vomiting, or occasionally sudden nasal flux, or (as at Walsall) " hæmorrhage from the nose, mouth, rectum, or all the mucous canals ;" the skin is hot and pungent ; the tongue thickly coated. The throat soon becomes painful, deglutition difficult, and considerable engorgement occurs of the submaxillary, parotid, and cervical glands. This characteristic engorgement increases to a surprising extent, the glands often projecting far beyond the jaw ; and the cellular tissues become deeply infiltrated and doughy to the touch. The throat, tonsils, and soft palate are covered with a yellow, leathery deposit, which exhales a fœtid odor that presently becomes intolerable. The patient is now in a condition of intense adynamia ; the pulse is rapid beyond limit, the face of vivid pallor, the lips congested, the eyes lachrymose, the mouth slobbering, deglutition difficult, perhaps almost impossible ; from the nostril often a fœtid ichor distils, showing that the exudation has also appeared on the walls of this cavity, where it may be seen if the nostril be expanded by an ear-speculum. Coma and extreme prostration follow ; and if a fatal termination ensue, the patient dies in a state of somnolent quietude which strongly contrasts wlth the agitation preceding croupal suffocation.

We need not to adduce examples of a type which has been but too commonly witnessed throughout the country. Many instances have been seen of an exceptional and much earlier termination by death.—Thus, at Bagshot, death occurred in many cases in the first stage through sudden and extreme adynamia, before the exudation had fully formed. Dr. Semple exhibited the parts in one such case at the Pathological Society; there was only a slight effusion into the larynx; the tonsils and neighboring parts were extensively congested, and it would appear that the patient had died from suffocation before there was time for further exudation to take place. Dr. C. J. B. Williams was called upon to treat cases in which the toxic influence had been equally apparent. Dr. Blount, of Bagshot, had added his experience in confirmation. In the fen district of Cambridge a series of cases occurred in May, in which death ensued from the intense depression of the vital powers.

The treatment of these cases must necessarily be energetic ; it needs to be essentially tonic. Of the various local applications, none equal in efficacy hydrochloric acid freely applied, or Beaufoy's concentrated solution of chloride of soda. This was used by Mr. Davey, of Rumford, in Essex; by Mr. Stiles, of Pinchbeck; by Mr. Wilkinson, of Spalding; by Dr. Cammack, of Spalding; and by many other practitioners who were seated amidst wide-spread epidemics, and their testimony is strongly in its favor. Where other local applications are employed, a gargle composed of two drachms of Beaufoy's solution to eight ounces of water should be used concurrently. Dr. Cammack recommends that two ounces of glycerine should be added to the gargle. When the nasal fossæ are implicated, such a solution should be injected through the nostril; it is preferable to the solution of alumen, or the insufflation of powdered alum, recommended by M. Bretonneau and M. Trousseau.

Of other local applications, alumen is too feeble; sulphate of copper not very efficient, and more poisonous; bromide of potassium, recommended by M. Ozaman* as a solvent of diphtheric exudation, theoretical and untried. Of the tincture of iodine, recommended by Dr. Marzel, we have no experience. Of the actual cautery, or of the cautery major dipped in boiling water, advised by Dr. Dauvin,† we do not desire to have any practical knowledge.

---

*Academic des Sciences, May 19th, 1856.
†L'Union Medicale, 1855.

Of the many internal remedies which have been advised, we do not know of any on which so much reliance can be placed as on the tincture of sesquichloride of iron, with chlorate of potass, chloric ether, and hydrochloric acid, in the form of mixture, sweetened with syrup, full doses being employed according to the age of the patient, and frequently repeated. A free use should be made of generous wine, beef-tea, coffee, eggs in combination with brandy and wine, milk, and whatever other form of nutriment the ingenuity of the surgeon or the fancy of the patient can suggest. When food is refused, then enemata similarly composed must be administered frequently, in small quantities of two ounces and upwards, that they may not be rejected; for it is of the first importance that inanition should not open the last portals of life to the advancing disease.

If the medical attendant should have the good fortune to vanquish the disease, he must be prepared to meet with long-enduring debility and adynamia—with a loathing for food, which will tax all his ingenuity and patience—and with various complications which call for a brief notice.

The most frequent sequence retarding convalescence is *paralysis of the soft palate.* This has been frequently noticed by various observers. The symptoms are, a nasal twang in the speech, incapacity for suction, and the regurgitation of fluids by the nostrils. The treatment is by local application of astringents, feeble cauterization, or by the employment of Duchenne's Faradization: one conductor to be applied to the soft palate, and the other over the mastoid process; or, as Duchenne himself recommends, both in one handle to the soft palate.‡ This paralysis may be of local origin; and Dr. Gull and Prof. Trousseau have held this opinion from cases under their observation. But a general paralysis has also frequently supervened, which is evidently the result of the general toxic influence of diphtheric poison on the blood. M. Trousseau had such a case lately under treatment at the Hotel Dieu, and it has induced him to change his opinion as to the local origin of the paralysis. Dr. Gull and Dr. Kingsford also relate instances. M. Herpin, the surgeon of Tours, was so affected during several months, and has given a graphic account of his symptoms.—Other sequences, more frequently observed in this country, have been severe otalgia, amaurosis and headache. Diphtheritic ophthalmia, of which epidemics were described in Germany, many by Græfe, in 1853-

‡See "L'Union Medicale," 1857, Trosseau and Lasegue: Dr. Moriceau, ibid. 1857; Dr. Maingault, "Theses de Paris," 1854, where an excellent monograph will be found on this subject.

54;* and by M. Jobert, at the Hôpital St. Eugenie, Paris, in 1857,† has been very little noted in this country.

In terminating this Report, we may offer some concluding propositions by way of summary:

I. Diphtheria is a *specific* disease. This is seen in its origin, march and mode of extension; in the character of its exudation; in its local manifestation; in its seats of predilection; in its toxic influence; in its prodromata; its manner of termination and its sequences.

II. It is often confounded with scarlatinal angina, and with gangrenous cynanche. We have sufficiently indicated the dsagnosis.

III. It is propagated by infection and by contagion. It is both epidemic and sporadic in its mannei of invasion, and is remarkable for the severity with which it is developed in limited centers of population.

IV. Diphtheric angina presents three varieties, which may be designated—1. Simple diphtheric angina. 2- Croupal diphtheric angina. 3. Malignant diphtheric angina. The prognosis of the first is favorable; of the second unfavorable; and of the third, most unfavorable.

V. The treatment should include the looal application of a solution of nitrate of silver, Beaufoy's concentrated solution of chloride of sodium, or hydrochloric acid, according to the circumstances also indicated. The internal remedies most useful are, emetics in the early stage of croupal diphtheria, and the tincture of sesqui-chloride of iron with chlorate of potash.

VI. Tracheotomy should be resorted to in the second or third stage of croupal diphtheria; leeching, blistering, and bleeding should always be avoided.

VII. The means of prevention, besides careful hygienic measures—as ventilation, etc.—must also include the daily examination of the throat where the epidemic type presides—a matter of the greatest importance, as experience has very fully shown, and the early insolation of the patient as soon as attacked—a precaution hardly less necessary.

*Note on Diphtheria.*

ARETÆUS, who lived neerly eighteen centuries ago, in his chapter *on ulcers of the tonsils*, has described a malignant variety of sore throat and mouth, which, to a great extent, agrees with those of the moderns, as being characteristic of malignant sore throat, diphtheria, black ton-

---

*Archives fur Ophthalmologie; see also "Gazette Hebdomadaire, h856, Warlomont et Test etin.

†Archives Generale de Med., 1857.

gue, membranous angina, etc. This malignant form has ulcerated surfaces, which "are broad, deep, foul, surrounded with a white, livid or black crust. The parts around the crust resemble carbuncle," etc.—He describes this disease as also involving the tongue, neck, trachea: "Patients thus affected," says he, "die in a few days of fever and inflammation, accompanied with fœtor, ichor, cough, dyspnœa, etc.—The mode of death is most miserable; there is a pungent, burning pain, like that of a carbuncle, with a fœtid breath—a putrid stench, which they reinspire into the chest, and so loathsome, that they cannot endure their own fœtor. They are hoarse, lose their voice," etc.

The history of epidemics for the last two or three centuries abounds with notices of this disease under the various names of malignant, strangulatory, pestilential, putrid, gagrenous sore throat, membranous croup, etc. These vague descriptions are not, however, accepted as diagnostic of diphtheria, as described more than thirty years ago by French pathologists, such as Gendrin, Bretonneau and others—a malady which is attended with a plastic or concrete membraniform exudation, and one occuring often among adults, generally as an epidemic, and supposed to be contagious.

The following note would seem to relate to diphtheria rather than to simple croup, as the former only has been regarded contagious.

B. D.

*Victim of Science.*—M. Sturne, health officer at Blaudeneques, near Saint Omer, porformed tracheotomy upon a young girl aged 16 years, who was attacked with croup; not having the canulas necessary in this operation conveniently at hand, he substituted for it a piece of elastic sound, which soon became clogged up. Mr. Sturne, in order to remove the obstacles, breathed through the tube by applying his mouth to its external extremity. This imprudent devotion was the cause of his loss. He was in turn attacked with croup, and in spite of the care of his neighboring confreres, in spite of tracheotomy, which was skilfully performed by Dr. Revil, the patient succumbed. M. Sturne leaves a widow and two children.—(*Translated from L'Union Médicale, by* S. E. C.)—*N. O. Med. & Surg. Journal.*

